# Effects of human immunoglobulins on *Cryptococcus neoformans* morphology and proteome

**DOI:** 10.1128/mbio.03827-25

**Published:** 2026-02-09

**Authors:** Taiane N. Souza, Haroldo C. de Oliveira, Antonio S. Nakouzi, Marlon D. M. Santos, Daniel Zamith-Miranda, Joshua D. Nosanchuk, Marcio L. Rodrigues, Liise-anne Pirofski

**Affiliations:** 1 Division of Infectious Diseases, Department of Medicine, Albert Einstein College of Medicine, Bronx, New York, USA; 2 Department of Microbiology and Immunology, Albert Einstein College of Medicine, Bronx, New York, USA; 3 Instituto Carlos Chagas, Fundação Oswaldo Cruz (Fiocruz) 169688 , Curitiba, Brazil; 4 Department of Microbiology, Immunology, and Parasitology, Discipline of Cellular Biology, Federal University of São Paulo (UNIFESP) 28105, São Paulo, Brazil; 5 Analytical Biochemistry and Proteomics Unit, Instituto de Investigaciones Biológicas Clemente Estable, Institut Pasteur de Montevideo, Montevideo, Uruguay; 6 Universidade Federal do Rio de Janeiro, Instituto de Microbiologia Paulo de Góes, Rio de Janeiro, Brazil; Universiteit Gent, Gent, Belgium

**Keywords:** *Cryptococcus neoformans*, human immunoglobulins, IgM, IgG, IgA, glucuronoxylomannan, cryptococcal capsule, cryptococcal proteome, cryptococcal meninigits, HIV-associated complications, cryptococcal pathogenesis

## Abstract

*Cryptococcus neoformans* (Cn) is the main cause of fungal meningitis in people living with HIV. Perturbations in normal immunoglobulin (Ig) levels are observed in these individuals, but their association with Cn pathogenesis is unclear. Here, we investigated the physical and biological effects of normal (not elicited by known cryptococcal infection) human immunoglobulins (Igs), IgM, IgG, and IgA on Cn (strain H99). Each isotype affected the growth, surface morphology, and proteome of Cn. However, IgA had the most prominent effect. It induced growth inhibition after 24 h of co-culture with Cn, altered the structural organization of capsular fibers, and significantly reduced protein synthesis and proteins associated with intracellular glucuronoxylomannan (GXM) synthesis, such as those mediating transport of sugar precursors to Golgi and the cyclic AMP pathway. Together with prior data showing an association between reduced plasma IgA and HIV-associated cryptococcal meningitis (CM), our findings suggest that the influence of human IgA on Cn pathogenesis warrants further investigation.

**IMPORTANCE:**

Cryptococcal meningitis (CM) causes approximately 1,200,000 deaths annually in people living with HIV and is also a threat to individuals with non-HIV-associated immune-compromising conditions, such as organ transplant recipients and other patients receiving immunosuppressants. Prior work has shown that normal human immunoglobulins (Igs) bind *Cryptococcus neoformans* (Cn) and that plasma levels of IgM, IgG, and IgA differ as a function of CM status. We investigated how human IgM, IgG, and IgA affect Cn growth, morphology, and protein synthesis. We found that IgA has major effects on these aspects of Cn biology and lends plausibility to the hypothesis that previously reported reductions in IgA levels in HIV-associated CM may influence Cn pathogenesis. Overall, our findings show that antibody immunity to Cn is more complex than previously thought.

## INTRODUCTION

*Cryptococcus neoformans* (Cn) is a cosmopolitan environmental fungus distinguished from other pathogenic fungi by a polysaccharide capsule. *Cryptococcus* species are responsible for the majority of fungal central nervous system infections, particularly meningitis, in immunocompromised individuals (1). A 2024 study estimated the global incidence of cryptococcal meningitis (CM) to be 194,000 cases resulting in 118,000 deaths (2). The development of CM may occur years after a primary lung infection due to reactivation of Cn in a latent state, most commonly in the setting of HIV-associated immunosuppression (3, 4). Resistance to CM in humans is primarily mediated by CD4^+^ T cells (5) and individuals with CD4 cell counts <200 cells/mm^3^ are at increased risk of reactivating Cn and developing CM. However, antibody immunity may also contribute to Cn reactivation, as perturbations in specific Ig binding to cryptococcal capsular polysaccharide glucuronoxylomannan (GXM) and plasma Ig levels have been identified in people with HIV with profound CD4^+^ T-cell deficiency, cryptococcal reactivation (cryptococcal antigenemia), and CM (6–10).

Normal human Igs bind to GXM, most likely reflecting serological memory from distant infection and latency (4, 11–13). These Igs are referred to as normal because they are not elicited by known Cn infection. In addition, the different states of cryptococcal infection—latent (asymptomatic), reactivation (antigenemia), and disease (CM) are associated with different Ig profiles (9, 10, 14, 15). A case-control study with HIV-infected individuals with and without CM showed reduced plasma IgG1, IgG2, and IgA levels in individuals with CM compared to those without CM (9). Relative reductions in IgM and IgA were also reported in another cohort of HIV-associated CM (14). On the other hand, increases in IgM (15) and IgG2 (10, 15) were reported in asymptomatic cryptococcal antigen-positive (CrAg^+^) HIV-infected patients, compared to those without HIV-associated cryptococcal antigenemia. Thus, levels of plasma Igs vary with the state of Cn infection in people with HIV.

Despite associations between human Ig levels and cryptococcal infection status in HIV-infected and HIV-uninfected people (7, 9, 10, 14, 15), the direct effect of human IgM, IgG, and IgA on Cn has not been rigorously characterized, although prior work showed that IgM and IgA, but not IgG, inhibit Cn Titan-like cell formation and alter Cn gene expression (16, 17). In the study presented herein, we investigated the effect of human IgM, IgG, and IgA on Cn growth, morphology, and proteome.

## RESULTS

### Human IgM, IgG, and IgA exhibit different patterns of binding to Cn

**Fig 1:**
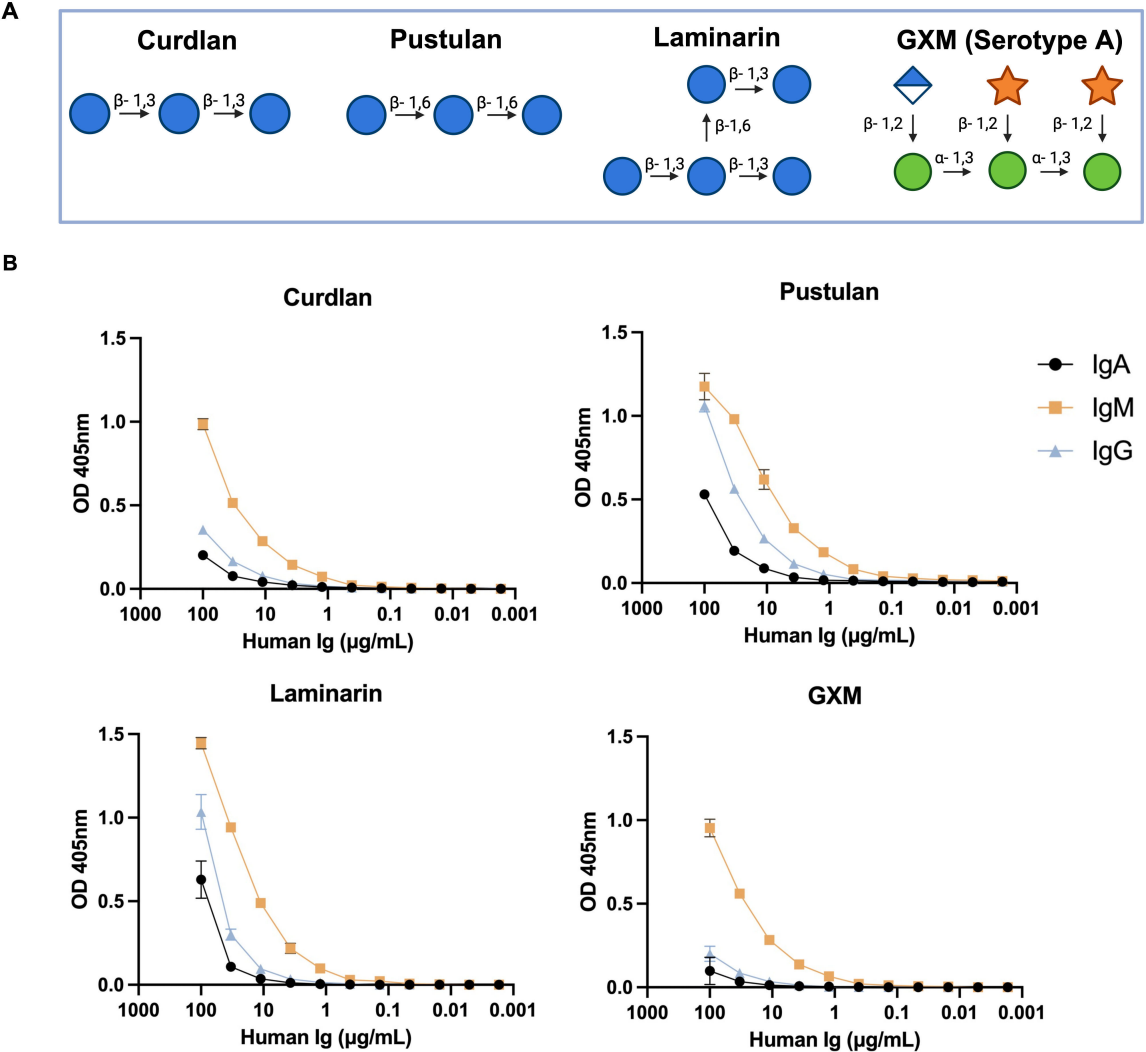
Human IgM, IgG, and IgA binding to glucan structures. (**A**) Schematic structures of β-glucans and GXM isolated from serotype A. (**B**) Binding of human IgM, IgG, and IgA to curdlan, pustulan, laminarin, and GXM assessed by ELISA. Graphs show the mean ± SD of absorbances representative of three independently performed experiments.

GXM is the main Cn capsular polysaccharide and virulence factor (18) and glucans are the most abundant polysaccharide in fungal cell walls (19). We evaluated binding of human IgM, IgG, and IgA to β-glucan structures: curdlan [β-(1, 3)-glucan], pustulan [β-(1, 6)-glucan], and laminarin [β-(1-3)-glucan with β-(1-6)-linkages] by ELISA (Fig. 1A). IgM had the highest reactivity to each β-glucan tested, while IgG and IgA binding were higher to pustulan and laminarin (Fig. 1B). IgA and IgG binding to curdlan were low but detectable. We also tested the reactivity of the Igs with GXM. All Ig exhibited measurable binding, but IgM reactivity was higher (Fig. 1B).

**Fig 2:**
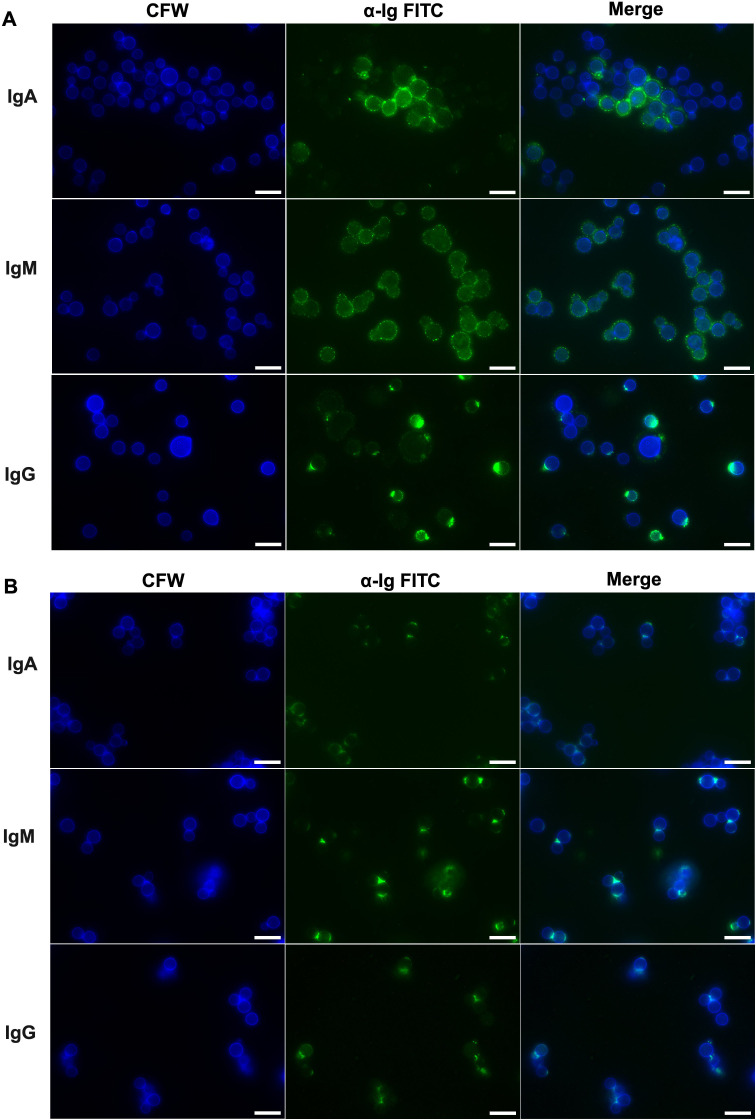
Human IgM, IgG, and IgA binding to Cn and *cap59*. Immunofluorescence staining performed as described in the text showing binding of IgA, IgM, and IgG to (**A**) Cn and (**B**) *cap59*. The cell wall was visualized by staining with Calcofluor white (CFW). BF, bright field. Scale bars, 10 μm. The images are representative of three independent experiments.

We then examined binding patterns of IgM, IgG, and IgA to the surface of live Cn and *cap59* by immunofluorescence (Fig. 2A and B, respectively). IgM and IgA binding were concentrated in the Cn surface (capsular) area, whereas IgG mainly colocalized in the cell wall area, with lower intensity binding to the capsular area (Fig. 2A). IgA fluorescence patterns were sparser and binding was observed to a smaller proportion of Cn compared to IgM and IgG (Fig. 2A). For *cap59*, IgM and IgA exhibited binding to the cell wall, as did IgG, which appeared to be concentrated in bud scar areas (Fig. 2B).

### IgA promotes complement-dependent phagocytosis of Cn

**Fig 3:**
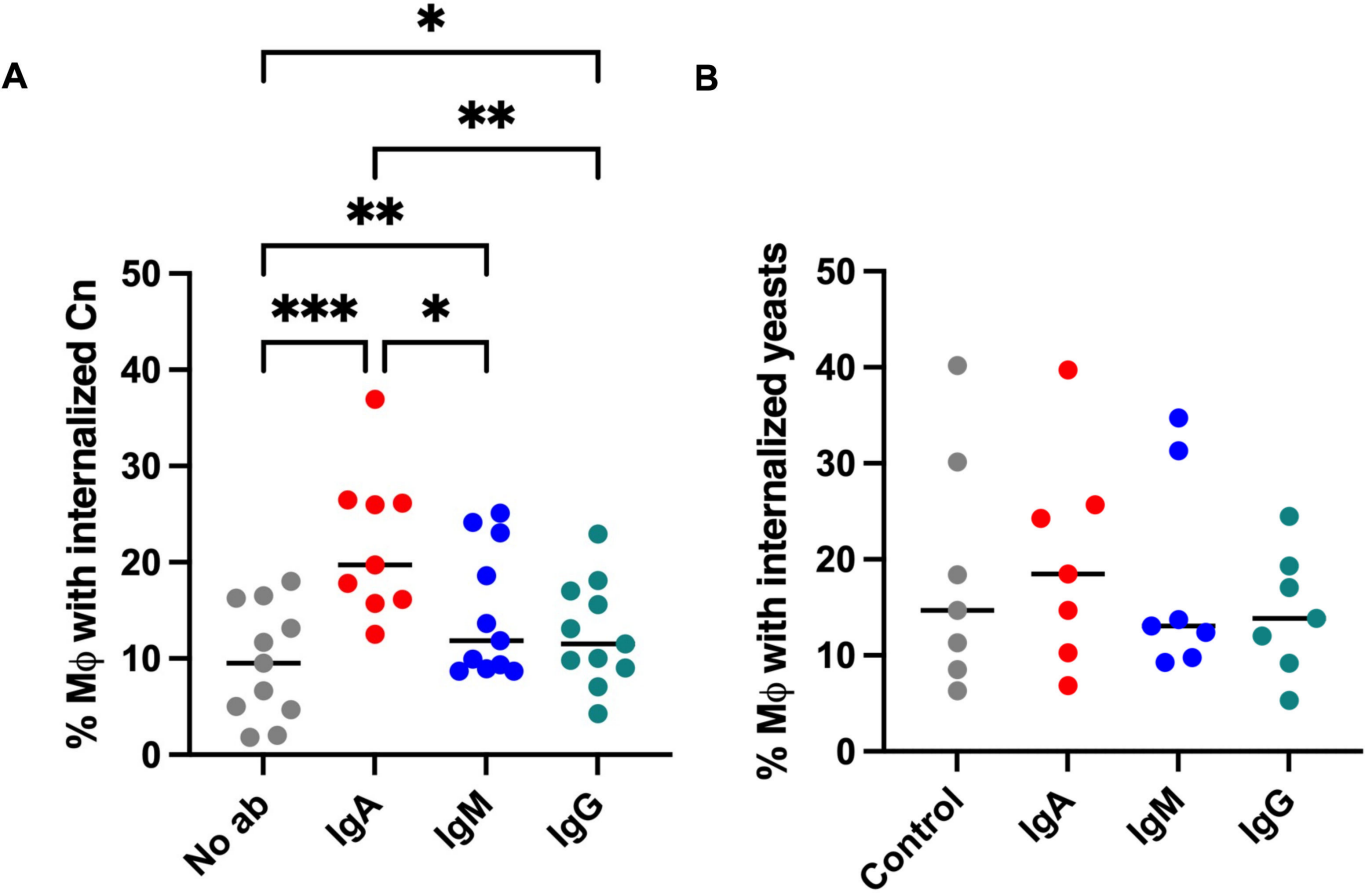
Effect of IgM, IgG, and IgA on complement-dependent phagocytosis of Cn by THP-1 cells. Graphs show the percentage of THP-1 cells with internalized (**A**) Cn and (**B**) *cap59*. Each dot represents the average of one experiment. Statistical analysis was performed using a one-way ANOVA matched pairs and a Bonferroni multiple-comparisons test. Graphs show means ± SD from at least seven independent experiments. *P < 0.05, **P < 0.03, and ***P < 0.001.

We evaluated the ability of IgM, IgG, and IgA to promote complement-dependent phagocytosis of Cn by the THP-1 cell line differentiated to macrophages. No phagocytosis was observed in the absence of complement (data not shown). With complement, IgA exhibited a statistically higher level of Cn uptake than PBS, IgM, or IgG (Fig. 3A). We also investigated internalization of *cap59* after incubation with the IgM, IgG, or IgA. No differences compared to the control were observed (Fig. 3B).

### IgM, IgG, and IgA delay Cn growth *in vitro*

**Fig 4:**
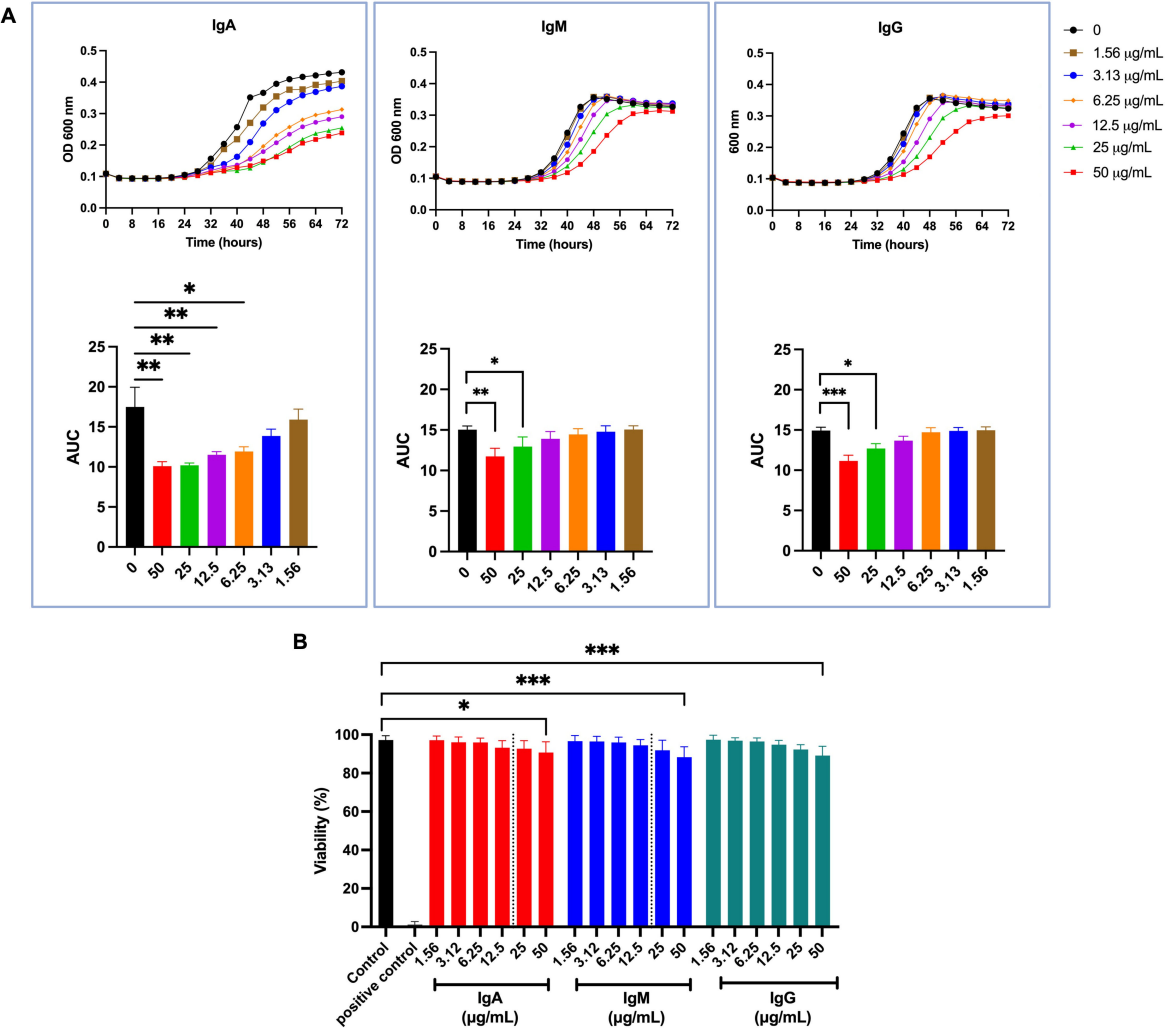
Effect of human IgM, IgG, and IgA on Cn growth. Cn was cultured in the presence or absence of different concentrations of IgA, IgM, or IgG in RPMI with MOPS for 72 h at 37°C as described in the text. (**A**) Growth curves of Cn depicted by optical density (OD 600 nm) on the *Y*-axis. The area under the curve (AUC) of each curve was calculated to perform the statistical analysis. (**B**) Viability of Cn determined by propidium iodide staining. Heat-killed Cn was used as a positive control. Statistical analysis was performed using a one-way ANOVA with Bonferroni multiple-comparisons test. Graphs show means ± SD from at least three independent experiments. *P < 0.05, **P < 0.01, and ***P < 0.001.

We examined the effect of IgM, IgG, and IgA on Cn growth and viability using MOPS-buffered RPMI media at 37°C after culture for 72 h. Growth kinetics showed that each isotype caused dose-dependent growth retardation (Fig. 4A). The area under the curve (AUC), which was used for statistical comparison of growth curves over time, showed significant inhibition of growth at 50, 25, and 12.5 µg/mL for IgA and at 50 and 25 µg/mL for IgM and IgG, compared to the control (Fig. 4A). To determine if growth delay reflected cell death, we assessed Cn viability with propidium iodide staining and quantified non-viable cells by flow cytometry. The highest concentration of each isotype tested (50 µg/mL) significantly reduced Cn viability by 10% (Fig. 4B).

### IgA affects capsule structure in Cn

**Fig 5:**
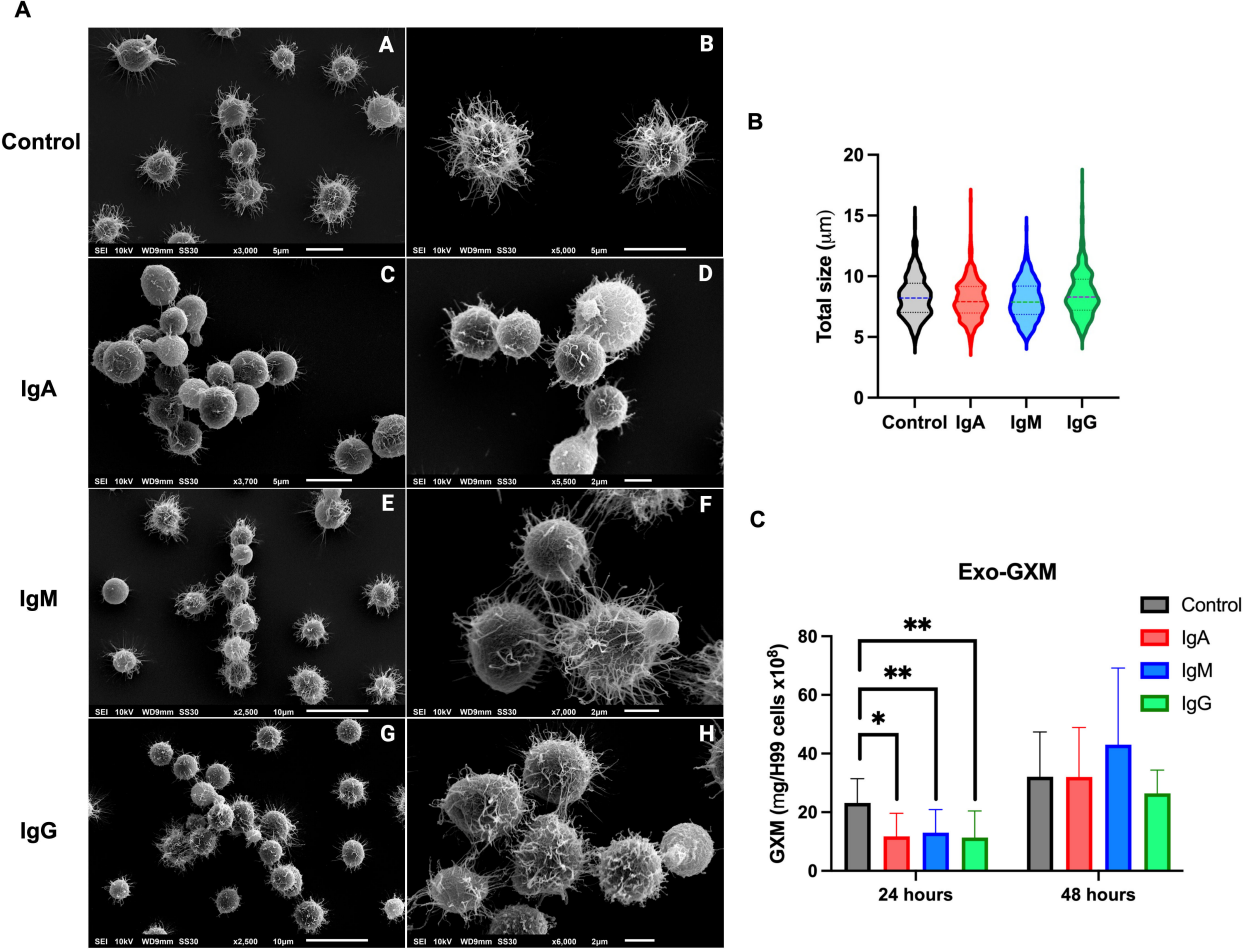
Effect of IgM, IgG, and IgA on Cn morphology. (**A**) Scanning electron microscopy of Cn incubated for 48 h with (**A and B**) PBS (control), (**C and D**) IgA, (**E and F**) IgM, and (**G and H**) IgG. (**B**) Total cellular size of Cn after 48 h and (**C**) concentrations of Cn GXM shed (exo-GXM) into culture supernatant after 24 and 48 h. Statistical analysis was performed using a one-way ANOVA matched pairs and a Bonferroni multiple-comparisons test. Graphs show means ± SD from at least three independent experiments. *P < 0.05 and **P < 0.03.

We examined the effect of IgM, IgG, and IgA on the surface of Cn by SEM. There was a change in capsular fiber organization after co-incubation with IgM, IgG, and IgA, as demonstrated by the cells having more compact capsular fibers compared to the control (Fig. 5A). However, there was a more pronounced effect with IgA, as capsular fibers were shorter and scarcer.

Quantification of Cn cell size by India ink staining revealed no differences compared to control after culture with IgM, IgG, or IgA (Fig. 5B). Analysis of extracellular GXM (exo-GXM) in supernatants of Cn cultured with each isotype revealed that each Ig significantly reduced shedding of GXM after 24 h (Fig. 5C). No differences in exo-GXM shedding between conditions were observed at 48 h. Collectively, these results demonstrated that co-culture of Cn with each isotype altered capsular structure and polysaccharide shedding.

### IgA impairs Cn protein synthesis

**Fig 6:**
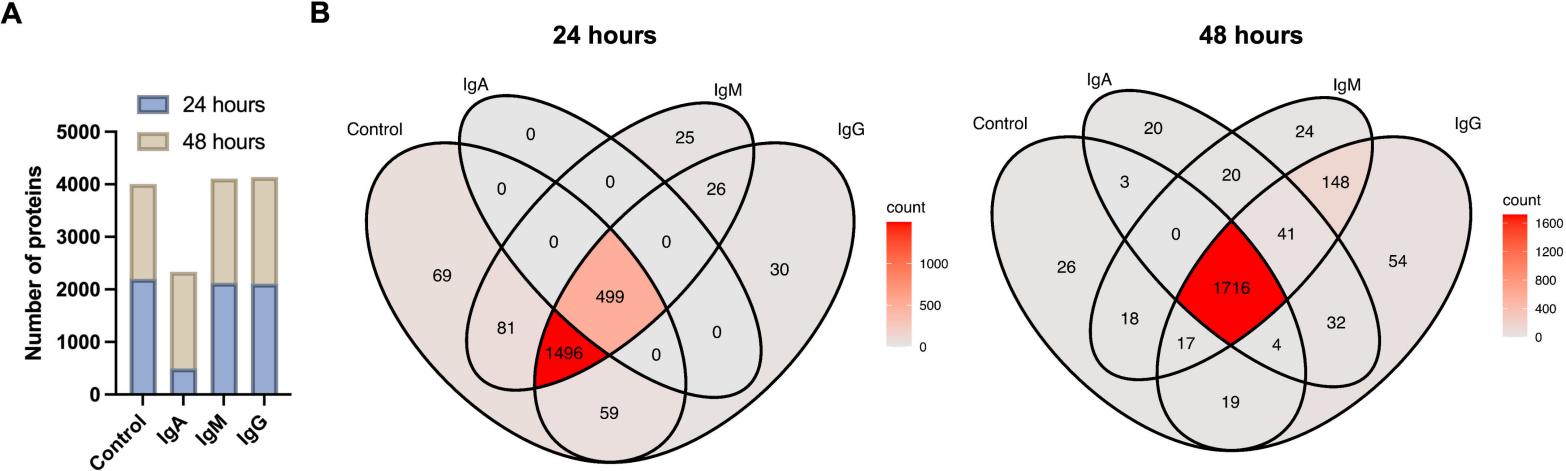
The effect of Cn cultured with IgM, IgG, and IgA on Cn protein synthesis. Cn proteins were isolated as described in the text. (**A**) Number of proteins identified in Cn cultured with IgM, IgG, IgA, and PBS (control) at 24 and 48 h. (**B**) Venn diagram of the comparative distribution of proteins for each isotype at 24 and 48 h. The scale depicts the range of protein quantities, from the fewest (light) to the most (darker red).

Proteomic analysis was performed to investigate the effects of co-culture of Cn with IgM, IgG, IgA, or control (PBS) on protein synthesis at 24 and 48 h. We detected 2,204 cellular proteins in the control group after 24 h of incubation. Most of these proteins (*n* = 1,496) were also found in Cn cultured with IgM or IgG, but not IgA (Fig. 6A and B). In Cn cultured with IgA, only 499 proteins were detected, all of which were also present in control, IgM, and IgG (Fig. 6A and B). After 48 h, the number of proteins detected in Cn cultured with IgA increased substantially, from 499 to 1,836 proteins (Fig. 6A and B). This increase consisted of proteins also present in other conditions (control, IgM, and IgG), and we identified 20 proteins unique to the IgA-treated group (Fig. 6B).

**Fig 7:**
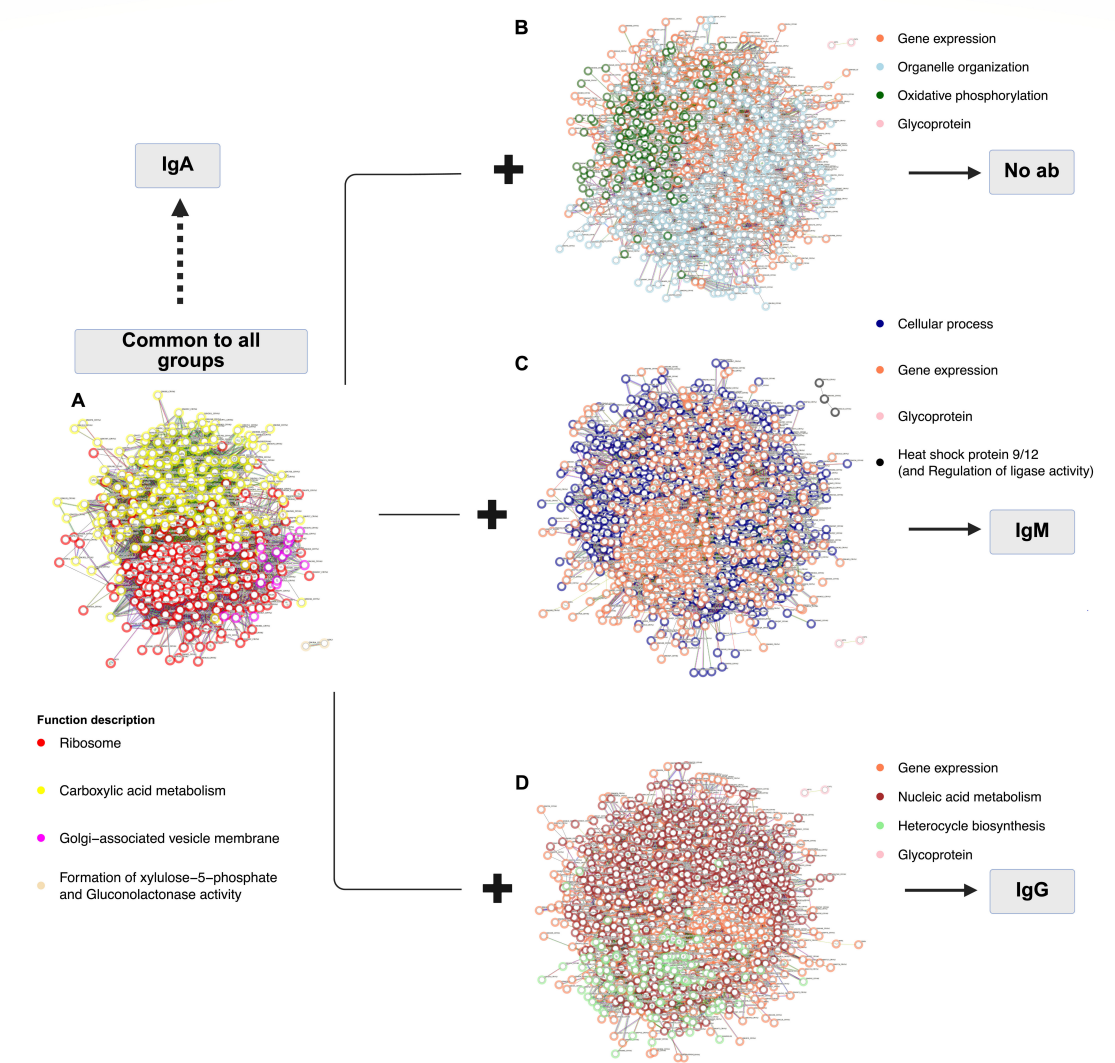
The effect of IgM, IgG, and IgA on protein-protein interaction (PPI) networks after 24 h of co-culture with Cn. PPI network analysis was performed using the STRING database. (**A**) Proteins in the proteome of all groups; control, IgM, IgG, and IgA had the same collection of proteins associated with the functions “Ribosome,” “Carboxylic acid metabolism,” “Golgi-associated vesicles membrane,” and “Formation of xylulose-5-phosphate and gluconolactonase activity.” PPI of additional proteins in (**B**) the control, (**C**) IgM, and (**D**) IgG.

The functional description of these proteins was analyzed by theoretical protein-protein interaction (PPI) network using the database STRING (Fig. 7). The PPI of the 499 proteins shared by all the groups (control, IgM IgG, and IgA) was associated with ribosome, carboxylic acid metabolism, and Golgi-associated vesicle membrane (Fig. 7A). The PPI of additional proteins present in the control group (1,705 proteins) was linked to gene expression, organelle organization, oxidative phosphorylation, and glycoprotein (Fig. 7B). Unlike the control, the PPI of additional proteins present in group IgM (1,628 proteins) was mainly associated with cellular processes (Fig. 7C), and in the group IgG (1,611 proteins) with nucleic acid metabolism and heterocycle biosynthesis (Fig. 7D). The function gene expression was also present in the PPI of IgM and IgG groups, as well as the function glycoprotein containing the two xylosylphosphotransferase-1, XPT1 and CXT1, involved in capsule biosynthesis.

Based on these findings, we searched for more capsule-associated proteins absent in Cn cultured with IgA for 24 h. Our analysis revealed the absence of key proteins involved in capsule biosynthesis, notably the UDP-galactose transporter and AMP-dependent protein kinase regulatory subunit (Table 1).

**TABLE 1:** Proteins related to capsule formation that were absent in Cn cultured with IgA for 24 h

ID	Description	Component
Q5KMJ4	UDP-glucose:glycoprotein glucosyltransferase	ER
Q5KE75	Hexose transporter	Membrane
Q5K8R6	Cryptococcal xylosyltransferase 1	Membrane
Q5K8Q8	UDP-galactose transporter	Golgi membrane
Q5KP84	Protein serine/threonine kinase, putative	Nucleus
Q5KNS1	cAMP-dependent protein kinase regulatory subunit	Nucleus
F5HAT2	Capsular-associated protein	Nuclear membrane
Q5KNC2	Capsular-associated protein	Membrane
Q5KMQ9	Mbp1 and Swi4-like APSES protein 1	Nuclear chromatin
Q5KMJ9	CAP64 gene product-related	Membrane
Q5KJY0	Capsular associated protein	Membrane
Q5KIN6	Vacuolar-ATPase subunit	Fungal-type vacuole membrane
Q5KIH2	Parallel beta-helix repeat protein	Membrane
Q5KIC0	Putative calcium-transporting ATPase	Fungal-type vacuole membrane
Q5KHS0	Transcription factor, putative	Nuclear chromatin
Q5KGF2	General transcriptional repressor, putative	Nucleus
Q5KF77	Rho GTPase-activating protein	Cytoplasm
P0CM46	pH-response regulator protein palA/RIM20	Endosome
P0CN96	Guanine nucleotide-binding protein subunit alpha	Cytoplasm
Q5K7C2	Protein kinase Sch9, putative	Fungal-type vacuolar membrane/nucleus
Q5K6X5	Transcription regulator, putative	Golgi apparatus/ER/membrane

**Fig 8:**
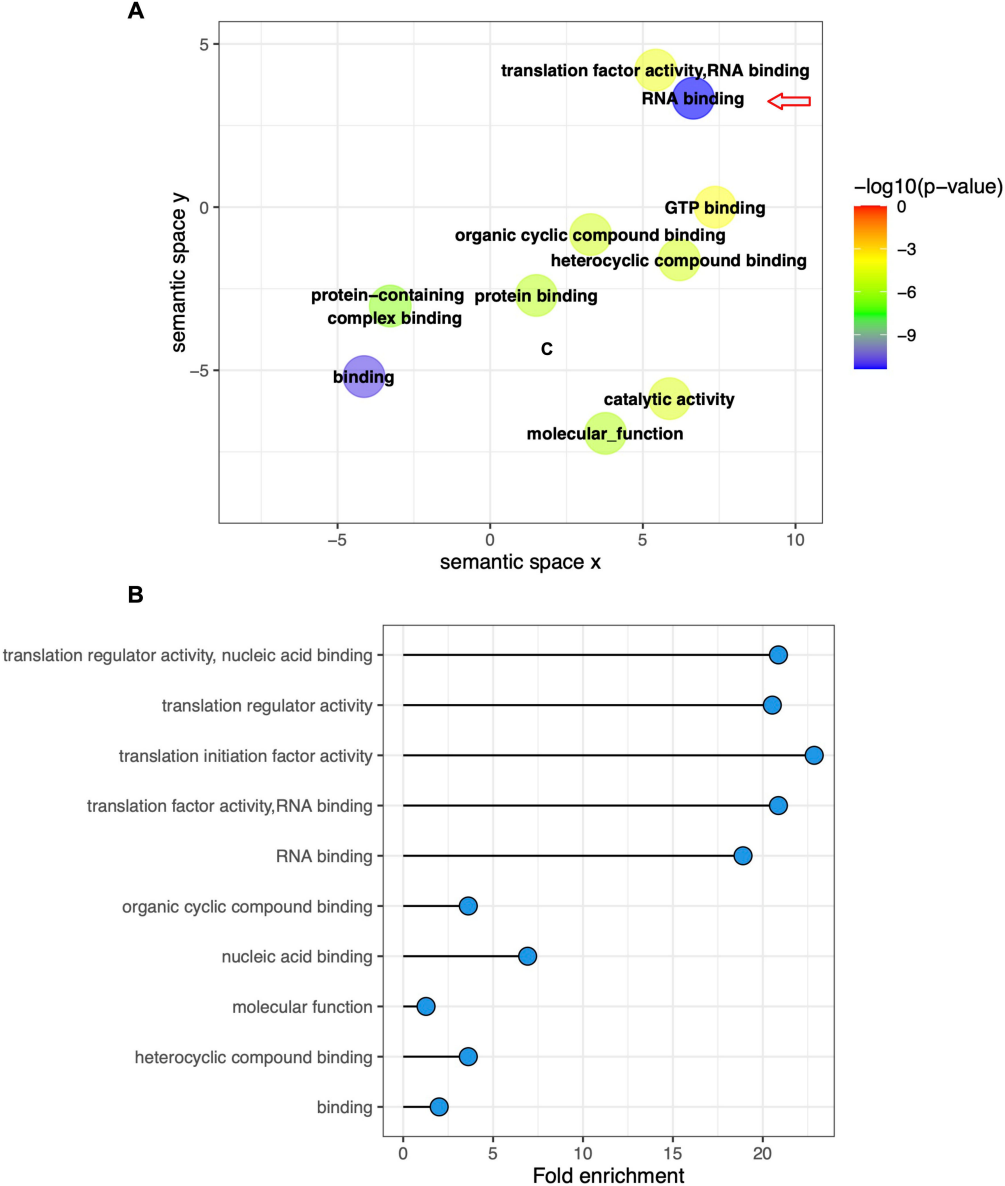
Cn proteins related to translation activity after co-culture with IgA for 24 h. Proteins were identified by functional enrichment analysis using FundiDB. (**A**) Bubble plot of the 10 GO terms most differentially expressed in Cn co-cultures with IgA, with the darker blue shading indicating higher significance from the Fisher exact test. RNA binding was the most significant term (arrow). (**B**) Plot of the 10 most significant protein functions enriched in the term RNA binding.

To identify more proteins that were absent in Cn cultured with IgA compared with control, IgM, and IgG for 24 h, we performed a gene ontology (GO) analysis using FungiDB to identify the function of the 1,496 proteins common to the control, IgM, and IgG, but absent in IgA. From this analysis, RNA binding proteins were the most frequent molecular class in this group (Fig. 8A). Subsequent analysis of the proteins associated with RNA binding revealed a significant enrichment of another term: translation activity-related molecules (Fig. 8B). Consistent with these findings, multiple translation initiation factors were not found in Cn cultured with IgA for 24 h (Table 2).

**TABLE 2:** Proteins related to translation activity in Cn after 24 h

ID	Description
Absent in Cn co-incubated with IgA	
Q5KHA2	Translation initiation factor
Q5KKV3	Eukaryotic translation initiation factor elF2B subunit alpha
Q5KC51	Eukaryotic translation initiation factor 2B alpha subunit
Q5K8D6	Eukaryotic translation initiation factor 2B subunit 2
Q5KB29	Eukaryotic translation initiation factor elF2B subunit gamma
Q5KE84	Eukaryotic translation initiation factor elF2B subunit delta
Q5KNW3	Eukaryotic translation initiation factor elF2B subunit epsilon
P0CN44	Eukaryotic translation initiation factor 3 subunit B
P0CN48	Eukaryotic translation initiation factor 3 subunit D
P0CN50	Eukaryotic translation initiation factor 3 subunit E
P0CO84	Eukaryotic translation initiation factor 3 subunit F
P0CN52	Eukaryotic translation initiation factor 3 subunit G
Q5KHC1	Eukaryotic translation initiation factor 3 subunit H
Q5KGC7	Eukaryotic translation initiation factor 3 subunit J
P0CN54	Eukaryotic translation initiation factor 3 subunit K
P0CN56	Eukaryotic translation initiation factor 3 subunit L
P0CM90	Eukaryotic translation initiation factor 3 subunit M
Q5K991	Translation initiation factor 4E
Q5KP78	Eukaryotic translation initiation factor 4F subunit P130
Q5KP94	Eukaryotic translation initiation factor 4F subunit P130
Q5KKF3	Cap binding protein
Q5KAS7	Eukaryotic translation initiation factor 6 (eif-6)
Present in Cn co-incubated with IgA	
P0CS32	Eukaryotic translation initiation factor 3 subunit I
Q5KIW8	Eukaryotic translation initiation factor 2 gamma
P0CN42	Eukaryotic translation initiation factor 3 110 kDa subunit (elf3p 110)
P0CN46	Eukaryotic translation initiation factor 3 subunit C
Q5K9Z0	RNA- binding protein sce3

**Fig 9:**
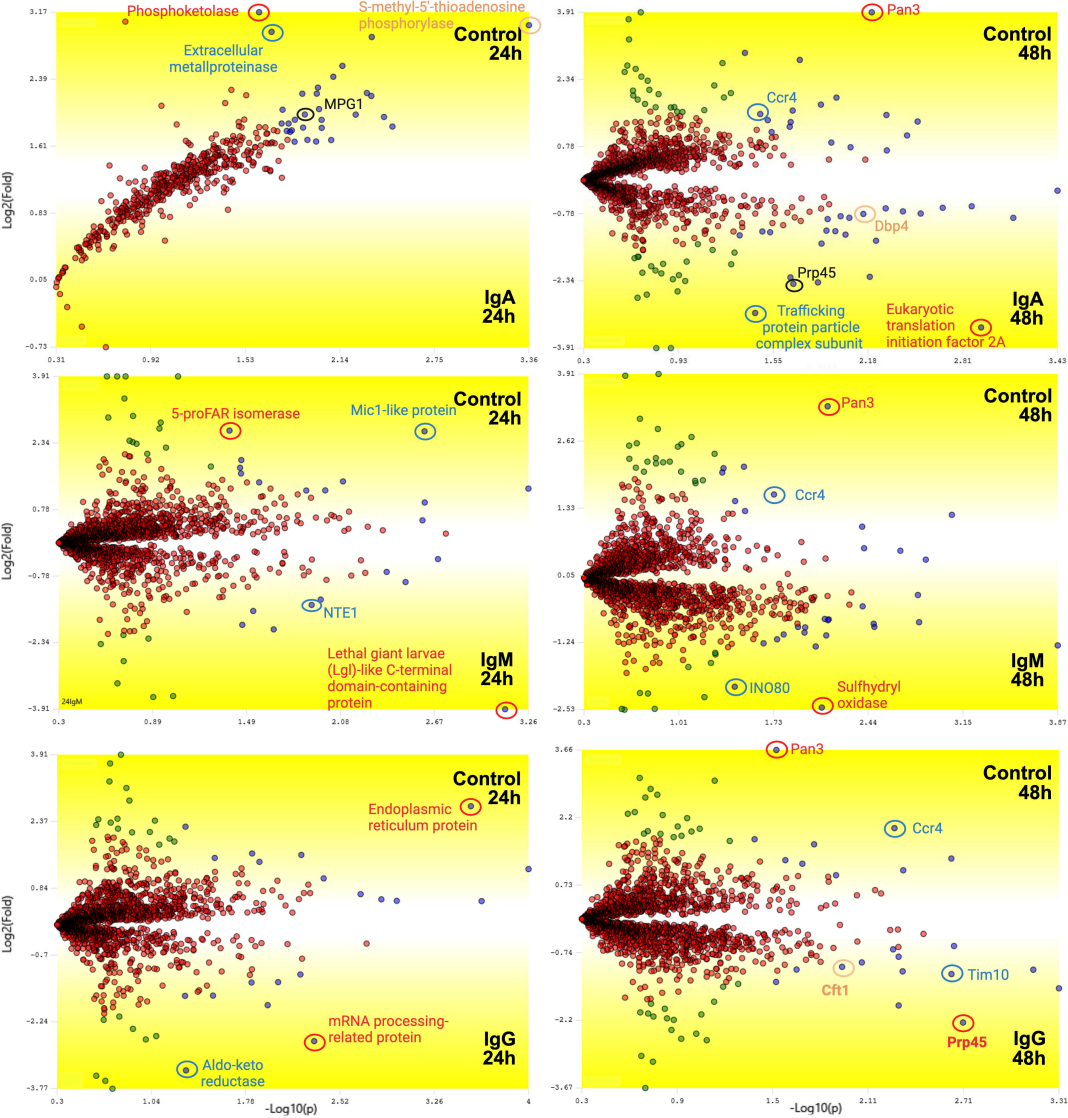
Differentially regulated Cn proteins after co-culture with human IgM, IgG, and IgA. Volcano plot generated using PatternLab’s TFold module, comparing Cn co-cultured with control to IgM, IgG, or IgA after 24 or 48 h. The software parameters used were a Benjamin Hochberg *q* value (FDR) of 0.05 and F-Stringency of 0.43. Each point represents a protein, mapped according to its −log_2_(*P* value) (*x*-axis) and log_2_(fold change) (*y*-axis). Red dots fall below the fold change and *q* value thresholds; green dots meet the fold change cutoff but not the *q* value cutoff. Blue dots satisfy both criteria, indicating statistically significant differential abundance.

Our next analysis was to identify proteins differentially regulated in Cn cultured with the Igs in comparison to the control (Fig. 9). After 24 h, no protein was significantly more abundant in Cn cultured with IgA. However, we detected downregulation of the protein MGP1, a mannose-1-phosphate guanyltransferase. In contrast, the most abundant proteins in cells co-cultured with IgM and IgG were the Lethal giant larvae-like C-terminal domain-containing protein and mRNA processing-related protein, respectively (Fig. 9). After 48 h, the statistical analysis revealed major changes in the proteome of Cn cultured with IgA, including a significantly increased abundance of the eukaryotic translation initiation factor 2A and trafficking protein particle complex subunit (Fig. 9). These findings align with our observation of increased protein synthesis in Cn cultured with IgA at 48 h. In Cn-IgM co-cultures, the enzymes sulfhydryl oxidase and INO80, a subunit of chromatin remodeling, were the most significantly overexpressed, while for Cn-IgG co-cultures the overexpressed proteins were the transcription-related proteins PRP45 and CFT1, and TIM10 (Fig. 9). In the control Ccr4 and Pan3 complexes, which have important roles in the growth on non-fermentable carbon sources, were the most predominant proteins in all comparisons.

## DISCUSSION

Host immunity is essential for the containment of Cn and resistance to CM. While T cell and innate responses have been extensively studied, less is known about the contribution of antibody immunity to cryptococcal resistance. Prior studies show that normal (not elicited by acute or known infection) human IgM and IgA inhibit a Cn virulence transformation, Titan-like cell induction, and alter Cn gene expression *in vitro* (16, 17). The present study provides a comprehensive analysis of the effects of human IgM, IgG, and IgA on Cn growth, morphology, and protein synthesis. Given that antigen-antibody reactions are inhibited in a pH range outside from 6.5 to 8.4, we chose RPMI medium buffered with MOPS to achieve a physiological pH, a medium that is used to evaluate antifungal drug susceptibility for clinical use (20).

Our immunofluorescence results show that human IgM and IgA bind primarily to the Cn capsule, while IgG bound mainly to the cell wall with the medium and growth conditions we used. The lack of observed binding of IgM and IgA to the Cn cell wall may be due to reduced access to cell wall epitopes, since their ability to bind such determinants is evident in their *cap59* binding. The larger size of these isotypes may limit their capacity to penetrate the capsule. Although precise identification of the Cn determinants targeted by each isotype will require dissecting individual specificities in polyclonal preparations, our data show IgM, IgG, and IgA target several distinct carbohydrate moieties, as evidenced by their binding to each β-glucan tested and GXM. IgA and IgG binding was most notable to pustulan and laminarin, suggesting these isotypes may have more restricted specificity for β-(1-6) glucans, perhaps for chitooligomers in the cell wall, which are enriched at sites of cell separation, budding sites, and bud scars. This aligns with the finding of Chiani et al. that normal human IgG exhibits more binding to pustulan than laminarin (21).

Human serum IgA binds galactose-α (Galα)-terminated glycans and sialoglycans in commensal bacteria (22). Since sialic acids linked to glycoproteins have been identified on the Cn cell wall (23), they may also be targets for IgA. Further work is needed to evaluate this possibility and to assess reactivity of IgA1 and IgA2 with Cn determinants. Further work is also needed to determine the reactivity of each isotype with galactose, a component of cryptococcal capsular glucuronoxylomannangalactan (GXMGal) (24), and other capsule-associated molecules, such as mannoproteins and lipids (25, 26). However, binding to isolated structures may not align with binding to fungal cells *in vivo*.

Mouse monoclonal antibodies that protect against lethal experimental cryptococcosis mediate many functions, including opsonization, growth inhibition, changes in capsule structure, inhibition of GXM release, and biofilm formation (27–32). Human GXM antibodies enhance monocyte phagocytosis of Cn and human GXM-IgG2 elicited by an experimental GXM vaccine mediated complement-independent phagocytosis of Cn by human monocytes (33). Our data show that IgA enhanced complement-dependent uptake of Cn by THP-1-derived macrophages. While surprising, because IgA demonstrated minimal binding to GXM by ELISA, it did bind the Cn surface by immunofluorescence. This may reflect the ability of polymeric IgA to enhance complement-mediated phagocytosis (34). Monomeric and polymeric IgA were not examined separately in this study.

Our data show that each Ig reduced the growth rate of Cn at higher concentrations, compared to the control, but viability was only reduced at a concentration of 50 µg/mL (or 0.05 mg/mL). Ig isotype concentrations in human serum are 1.47 mg/mL for IgM, 2.62 mg/mL for IgA, and 11.18 mg/mL for IgG (35). While it is not possible to extrapolate from *in vitro* conditions in the laboratory to *in vivo* in the human body, our data suggest the hypothesis that human Ig might affect Cn growth and viability in people is biologically plausible. We also observed that each Ig isotype reduced exo-GXM shedding compared to the control and affected Cn capsular morphology, with IgA having the most dramatic effect by scanning electron microscopy (SEM). Although we did not quantify capsular size of IgA-treated cells, our findings align with prior data that showed IgA reduced Cn cell size and inhibited Titan-like cell formation (capsular enlargement) when cultured in Sabouraud and Titan Cell Medium (16, 17).

Although we do not know the mechanism by which IgA affects capsule structure, our proteomic data identified several proteins that may be involved in these changes. When Cn was co-cultured with IgA for 24 h, there was a reduction in protein synthesis resulting in a smaller overall number of proteins and translation initiation factors compared to IgM, IgG, and PBS. Notably, there was a reduced number of proteins related to polymerization of capsule precursors via Golgi apparatus, such as cryptococcal xylosiltransferase 1 and UDP-galactose transporter. UDP-galactose reaches the Golgi lumen through the UDP-galactose transporter, which is important for GXMGal biosynthesis (36). Cryptococcal xylosiltransferase 1 is a glycosyltransferase that transfers xylose to GXM and GXMGal (37). Deletion of either of these proteins reduced the virulence of Cn i*n vitro* and *in vivo* (36, 38).

Another protein absent in cells co-cultured with IgA was the cyclic AMP-dependent protein kinase regulatory subunit, a protein associated with an important signaling pathway involved in the formation of Cn capsule (39). The deletion of the PKA1 gene encoding for this protein affects capsule formation and reduces virulence (39). The absence of these proteins in Cn co-cultured with IgA suggests that unlike IgM and IgG, IgA may affect the first steps of GXM synthesis. This aligns with the prominent effect of IgA on Cn capsule morphology.

Although most of the proteomic changes induced by the Ig isotypes were observed within 24 h of co-culture, there was a downregulation of the cytoplasmic deadenylase complexes Ccr4-Not and Pan2-Pan3 with each isotype after 48 h. Pan2-Pan3 complexes and Ccr4-Not complex regulate the length of poly(A) tail in mRNA of fungi, and both deadenylases are important for Cn growth and adaptation to stress (40–42). For instance, Ccr4-Not complex was responsible for a rapid post-transcriptional regulation of genes in adaptation to host temperature at 37°C. A Cn Ccr4 mutant exhibited reduced growth, increased exposure of cell wall α and β-glucans, enhanced recognition by dectin-1, and increased phagocytosis by lung macrophages (41). This isotype-independent effect aligns with the slower growth of Cn when co-cultured with each isotype tested, suggesting that they may similarly impact the Cn stress response.

Collectively, our data demonstrate that co-culture of human IgM, IgG, and IgA with Cn induced multiple changes ranging from effects on growth, capsular morphology, and protein synthesis, which were most notable for IgA. However, our study has numerous limitations. We only used one strain of Cn (H99). Our findings need to be extended to clinical serotype A strains and other cryptococcus species, but these studies were beyond the scope of this study. Cn virulence and biology are affected by culture conditions (43, 44). We only used one, albeit physiological pH growth condition without capsule induction. We observed binding of each Ig isotype to Cn but did not study monomeric and dimeric IgA separately or identify the capsular and cell wall targets of the polyclonal preparations. This work was beyond the resources and scope of this study. Nonetheless, our data are novel and suggest that Cn-binding antibodies in human serum may influence Cn physiology and virulence. Our findings call for further research to identify the Cn epitopes targeted by each isotype and in-depth mechanistic studies to determine how the Ig isotypes affect Cn growth and morphology, especially IgA. Finally, the marked effects of human IgA on Cn described in this study, along with prior data associating lower IgA levels with CM (9), suggest that investigation of innate and mucosal immune responses to Cn may advance our understanding of Cn pathogenesis and inform the development of immunotherapeutic approaches to improve CM outcomes.

## MATERIALS AND METHODS

### Reagents and cryptococcal strains

#### Human immunoglobulins (Igs)

Human IgM, IgG, and IgA isolated from human serum (Sigma, catalog numbers, I4036, I8260 and I4506, respectively) were dialyzed to remove sodium azide (Slide-A-Lyzer Dialysis Cassettes, 7K MWCO, Thermo Scientific).

#### Carbohydrates

Antigens were obtained from commercial sources. Laminarin, a complex polysaccharide consisting of linear β-(1-3) and β-(1-6) glucan branches (Sigma), and pustulan, a linear β-(1-6) glucan (InvivoGen), were solubilized in PBS. Curdlan, a linear β-(1-3) glucan (Sigma), was solubilized in 0.1 N sodium hydroxide, then neutralized with hydrochloric acid (HCl) to achieve a neutral pH. GXM was isolated from serotype A Cn strain H99 as described in reference 45.

#### Cryptococcal strains

*C. neoformans* var. *grubii* strain H99 (H99), serotype A, was used in all experiments. This strain is used extensively in studies of cryptococcal biology and pathogenesis (46). The mutant acapsular strain *cap59,* derived from parent background H99 and an mCherry-expressing H99 strain, was kindly provided by Arturo Casadevall (Johns Hopkins School of Public Health).

### Cell line

The human cell line THP-1 (human monocyte) was obtained from ATCC (American Type Culture Collection, TIB-202).

### Complement

Baby rabbit complement was obtained from Pel-Freez Biologicals.

### ELISA for determination of carbohydrate-binding Igs

Binding of human IgM, IgG, IgA to laminarin, GXM, pustulan, and curdlan, was determined by ELISA as previously described (16, 47). Briefly, high-binding ELISA plates (Costar) were coated (50 μL/well) with 100 μg/mL of laminarin, pustulan, curdlan, or GXM and incubated overnight at 4°C. Plates were then blocked with 1% (wt/vol) BSA (Sigma) in PBS (200 μL/well) and incubated for 1 h at 37°C. Carbohydrate-coated plates were incubated with serial threefold dilutions of human IgM, IgG, or IgA (starting with 100 μg/mL of Ig diluted in blocking buffer, 50 μL/well) and incubated for 1 h at 37°C. After washing three times with PBS containing 0.05% of Tween-20 (vol/vol) using an AquaMax 2000 microplate washer (Molecular Devices), plates were incubated with anti-human IgM, IgG, or IgA antibodies conjugated to alkaline phosphatase (AP) (Southern Biotech) for 1 h at 37°C. The plates were washed, incubated with p-nitrophenyl phosphate (PNPP) at room temperature (RT), and the resulting color was measured by a SpectraMax ABS reader (Molecular Devices) at 405 nm.

### Growth of Cn with human Igs

Cn var. *grubii* H99 cells (heretofore Cn) or acapsular mutant *cap59* (heretofore *cap59*) were cultured in Sabouraud (BD Biosciences) broth for 48 h at 30°C in a rotatory shaker at 150 rpm and seeded onto Sabouraud agar plates. For experiments, one colony was inoculated into Sabouraud broth and grown under the same conditions for 24 h. Then, yeasts were washed three times with PBS and inoculated into modified RPMI (RPMI 1640 medium (Sigma R1383) with 2% glucose, buffered with 0.165 M MOPS and adjusted to pH 7), containing (or not) human IgM, IgG, IgA (Sigma) and incubated at 37°C.

### Cn immunofluorescence with human Igs

Cn or *cap59* (10^7^ cells) were incubated with human IgM, IgG, IgA (100 μg/mL in PBS) or PBS in Pierce Immunostain Enhancer solution (Thermo Fisher Scientific Inc.) for 1 h at 37°C. Cells were washed three times with PBS and stained with FITC-conjugated goat anti-human IgM, IgG, IgA (Southern Biotech) at 10 μg/mL in Immunostain Enhancer for 1 h at 37°C, and Calcofluor White (chitin stain) for 5 min at RT in the dark. The cells were washed twice and mounted in 50 mM n-propyl gallate in PBS. The slides were visualized using a Carl Zeiss Imager.Z1.

### Growth kinetics and Cn viability

Growth kinetics assays were performed using broth microdilution in accordance with Clinical and Laboratory Standards Institute, with minor modifications. Cn (5 × 10^4^ cells/mL) in MOPS-buffered RPMI medium were incubated in a 96-well plate with concentrations of IgM, IgG, IgA, ranging from 1.56 to 50 µg/mL, or PBS as a control. Plates were incubated at 37°C for 4 days, with continuous shaking, and the absorbance measured hourly at 600 nm. Growth was evaluated statistically by comparing the AUC of experimental groups to the control. To assess Cn viability, cells were stained at day 4 with 10 μg/mL of propidium iodide. Fluorescence intensity was measured by flow cytometry using a FACSCalibur cytometer (Becton Dickinson) and analyzed on software FlowJo (BD Biosciences). Heat-killed and viable cells were included as controls.

### THP-1 culture, differentiation to macrophages, and phagocytosis assay

The ability of IgM, IgG, IgA to promote phagocytosis of Cn was determined using human THP-1 cells. The cells were cultured in suspension in high glucose RPMI medium (ATCC), supplemented with 10% (vol/vol) FBS (R&D Systems) and 1% penicillin/streptomycin (Corning), at 37°C and 5% CO_2_. Differentiation of THP-1 cells to macrophages was induced as follows: 5 × 10^5^ THP-1 cells/mL were plated in a Nunc Lab-Tek II chamber slide system (four-well chamber slide) with 200 nM phorbol 12-myristiate-12 acetate (PMA, Sigma) for 48 h, followed by 24 h incubation in PMA-free RPMI medium.

Phagocytosis of Cn was determined as follows: a mCherry-expressing Cn or *cap59* pre-stained, with 10 mM of FUN-1 (Thermo Fisher Scientific Inc.) for 45 min at 30°C, were incubated for 1 h at 37°C with 25 μg/mL of IgM, IgG, IgA, or PBS (control), in the presence or absence of 20% complement (3–4 weeks rabbit complement, Pel Freez). Opsonized yeasts were added to THP-1 macrophages at a 1:1 multiplicity of infection and incubated for 2 h at 37°C. Following three washes with PBS, the cells were counterstained with 0.2 g/L Calcofluor White (5 min at 25°C) to distinguish internalized from non-internalized yeasts. Finally, the cells were washed three times with PBS, fixed with a 2% formaldehyde solution in PBS, and analyzed by fluorescence microscopy. For the microscopic analysis, at least 200 cells per group were counted to determine the percentage of phagocytosis and phagocytic index. The percentage of phagocytosis was calculated according to the following formula: (number of macrophages containing engulfed yeasts) divided by (total number of macrophages).

### Analysis of Cn capsule structure

The effect of IgM, IgG, and IgA on the morphology of Cn was examined using light microscopy with India ink staining and SEM. Cn (10^5^ cells/mL) were incubated or not with IgM, IgG, or IgA (25 µg/mL) for 48 h in MOPS-buffered RPMI medium at 37°C. For SEM, yeasts were washed with PBS, fixed with 2.5% glutaraldehyde in 0.1 M sodium cacodylate buffer (pH 7.2) for 1 h at RT, washed with a 0.1 M sodium cacodylate buffer (pH 7.2) containing 0.2 M sucrose and 2 mM MgCl_2_, and then placed over 0.01% poly-l-lysine-coated coverslips and incubated for 30 min at RT. Adhered cells were then gradually dehydrated in 30%, 50%, and 70% ethanol for 5 min, 90% for 10 min, and then 100% twice for 10 min. Immediately after dehydration, the cells were critical point dried (Leica EM CPD300), mounted on metallic bases, and coated with a gold layer (Leica EM ACE200). Yeasts were then visualized with a scanning electron microscope (JEOL JSM-6010 Plus/LA) operating at 10 kV. For India ink staining, 5 μL of 48-h Cn suspension was mixed with an India ink drop (India ink reagent droppers; BD Biosciences) on a microscope slide. Samples were examined using a Zeiss AXIO Imager.Z1 microscope, with at least five random fields photographed at 63× magnification. Cellular dimensions were measured for at least 100 individual yeasts from digitalized images using ImageJ software (48).

### Proteomic analysis

Cn (10^5^ cells/mL) were incubated with IgM, IgG, or IgA (25 µg/mL) or PBS (control) for 24 (one set of cultures) or 48 h (another set of cultures) at 37°C with continuous agitation (150 rpm). Proteins were extracted, prepared, identified, and analyzed as previously described (49). Briefly, the cells were washed with PBS, collected, and the pellets were frozen in an ultra-freezer (−80°C). Pellets were then lyophilized and lysed with 0.1 mm zirconium beads. Subsequently, a 0.1% RapiGest SF solution (wt/vol) in 50 mM ammonium bicarbonate (NH_4_HCO_3_) was used to suspend the protein lysates. After centrifugation at 18,000 × *g* for 15 min, the supernatants were collected, and the protein concentration was determined by fluorometric quantification (Qubit, Thermo Fisher Scientific Inc.).

Isolated peptides were loaded onto in-house packed reverse-phase (75 μm × 30 cm) with ReprosilPur C18 Acqua stationary phase (3 μm diameter beads and 120 Å pore size, Dr. Maisch) in a nanochromatography system, Ultimate 3000 (Thermo Fisher Scientific Inc.). A 120-min gradient (5–40% phase B: 95% acetonitrile/0.1% formic acid) eluted peptides were collected into an Orbitrap Fusion Lumos mass spectrometer (Thermo Fisher Scientific Inc.) at the Proteomics and Mass Spectrometry facility at the Carlos Chagas Institute, Fiocruz-PR. Data were acquired in data-dependent acquisition mode (60,000 MS1 resolution; 15,000 MS2, both at 200 *m/z*) with a 2 s cycle time, selecting the top ions (excluding unassigned charge states and ions with a 1+ charge state) for higher-energy collisional dissociation fragmentation (30% normalized collision energy [NCE]). Dynamic exclusion was set to 60 s. Additional parameters: 2.6 kV spray voltage, 250°C capillary, zero flow of sheath/auxiliary gas, predictive automatic gain control, and 70% S-lens radiofrequency (RF). Xcalibur 4.0 controlled all workflows (Thermo Fisher Scientific).

Proteomic data were analyzed using PatternLab for Proteomics V (50) with Y.A.D.A. 3.0 for multiplexed spectra identification (51). A Cn protein database UniProt (http://www.uniprot.org), accessed on 27 January 2024, was compiled using the target-decoy approach and included reversed sequences and 127 common contaminants (e.g., keratin, and trypsin). The Comet search tool (52) was used to compare the experimentally obtained tandem mass spectra with the theoretical spectra generated from the database. The search was limited to tryptic candidates with up to two cleavage failures, and carbamidomethylation of cysteine was considered a fixed modification and oxidation of methionine was considered a variable modification. Validation was performed using the Search Engine Processor (SEPro) and relative protein quantification was performed as previously described (49). The FungiDB database (https://fungidb.org/fungidb/app) was used to categorize the proteins by GO annotation (53). The STRING database (https://string-db.org/) was used to integrate PPIs (54).

## Data Availability

The mass spectrometry proteomics data have been deposited to the ProteomeXchange Consortium via the PRIDE (55) partner repository with the data set identifier PXD072710 (https://www.ebi.ac.uk/pride/).

## SUPPLEMENTAL MATERIAL

Captions mbio.03827-25-s0001.docx Supplemental material captions.

Table S1 mbio.03827-25-s0002.xlsx Protein distribution in the experimental groups at 24 hours.

Table S2 mbio.03827-25-s0003.xlsx Protein distribution in the experimental groups at 48 hours.

Table S3, part 1 mbio.03827-25-s0004.xlsx Proteins with significantly altered abundance in the experimental groups at 24 hours.

Table S3, part 2 mbio.03827-25-s0005.xlsx Proteins with significantly altered abundance in the experimental groups at 48 hours.

ASM does not own the copyrights to Supplemental Material that may be linked to, or accessed through, an article. The authors have granted ASM a non-exclusive, world-wide license to publish the Supplemental Material files. Please contact the corresponding author directly for reuse.
