# Supplementary material for: Effects of human immunoglobulins on Cryptococcus neoformans morphology and proteome
Source: mBio. 2026 Feb 9;17(3):e03827-25. doi: 10.1128/mbio.03827-25 (PMC12977590; doi:10.1128/mbio.03827-25)
Supplement: Captions — Supplemental material captions. [file mbio.03827-25-s0001.docx]

**SUPPLEMENTAL MATERIAL LEGENDS.**

**Supplemental Table S1:** Venn analysis at 24 hours. List of proteins that are exclusive to or shared among the control group and *C. neoformans* (Cn) cultured with IgM, IgG, or IgA.

**Supplemental Table S2:** Venn analysis at 48 hours. List of proteins that are exclusive to or shared among the control group and *C. neoformans* (Cn) cultured with IgM, IgG, or IgA.

**Supplemental Table S3:** Volcano plot analysis at 24 hours. List of proteins with significantly altered abundance in *C. neoformans* (Cn) following 24-hour culture with IgM, IgG, or IgA compared to the control.

**Supplemental Table S3:** Volcano plot analysis at 48 hours. List of proteins with significantly altered abundance in *C. neoformans* (Cn) following 48-hour culture with IgM, IgG, or IgA compared to the control.
